# Human chorionic gonadotropin dose response for induction of ovulation 7 days after a synchronized ovulation in lactating Holstein cows

**DOI:** 10.3168/jdsc.2020-0024

**Published:** 2020-12-11

**Authors:** E.M. Cabrera, M.R. Lauber, E.M. Peralta, T.R. Bilby, P.M. Fricke

**Affiliations:** 1 Department of Animal and Dairy Sciences, University of Wisconsin-Madison, Madison 53706; 2 Merck Animal Health, Kenilworth, NJ 07033

## Abstract

Our objective was to assess the effect of treatment with GnRH or 4 increasing doses of human chorionic gonadotropin (hCG) on the ovulatory response of a first-wave dominant follicle and subsequent plasma progesterone (P4) concentrations. Lactating Holstein cows were blocked by parity (primiparous vs. multiparous) and randomly assigned to receive no treatment (control, CON; n = 147), 100 μg of GnRH (n = 144), or 1,000 (n = 138), 2,000 (n = 144), 2,500 (n = 142), or 3,300 (n = 139) IU of hCG 7 d after the last GnRH treatment (G2) of a Double-Ovsynch (DO) or Resynch protocol. Blood samples were collected and ovaries were evaluated with transrectal ultrasonography immediately before treatment and 7 d later to assess serum P4 concentrations and ovulatory response to treatment. Data were analyzed using the MIXED and GLIMMIX procedures of SAS (SAS Institute Inc., Cary, NC). Overall, ovulatory response differed and was 4.8, 79.0, 77.4, 88.9, 92.9, and 95.6% for CON, GnRH, 1,000-, 2,000-, 2,500-, and 3,300-IU hCG treatments, respectively. The increase in plasma P4 concentrations from 7 to 14 d after G2 differed among treatments and was 3.5, 5.9, 5.7, 6.6, 7.0, and 6.5 ng/mL for CON, GnRH, 1,000-, 2,000-, 2,500-, and 3,300-IU hCG treatments, respectively. In conclusion, lactating Holstein cows treated 7 d after G2 with 100 μg of GnRH or 1,000 IU of hCG had similar ovulatory responses (~78%), whereas cows treated with 2,000, 2,500, or 3,300 IU of hCG had increased ovulatory responses (~92%). Ovulatory response of cows treated with 2,000 or 2,500 IU of hCG did not differ, whereas the ovulatory response after 3,300 IU was greater than that after 2,000 IU of hCG. Plasma P4 concentrations and luteal volume 7 d after treatment were increased compared with those of untreated control cows.

## Highlights

• Doses of hCG evaluated in published experiments range from 1,000 to 3,300 IU
• The optimal dose of hCG to induce ovulation on day 7 of the estrous cycle in lactating Holsteins was 2,500 IU
• The greatest increase in progesterone concentration from day 7 to 14 resulted from the 2,500 IU hCG dose

## Graphical Abstract

Effect of treatment with gonadotropin-releading hormone (GnRH)or 4 doses of human chorionic gonadotropin (hCG)on ovulatory response in lactating Holstein cows

**Figure:**
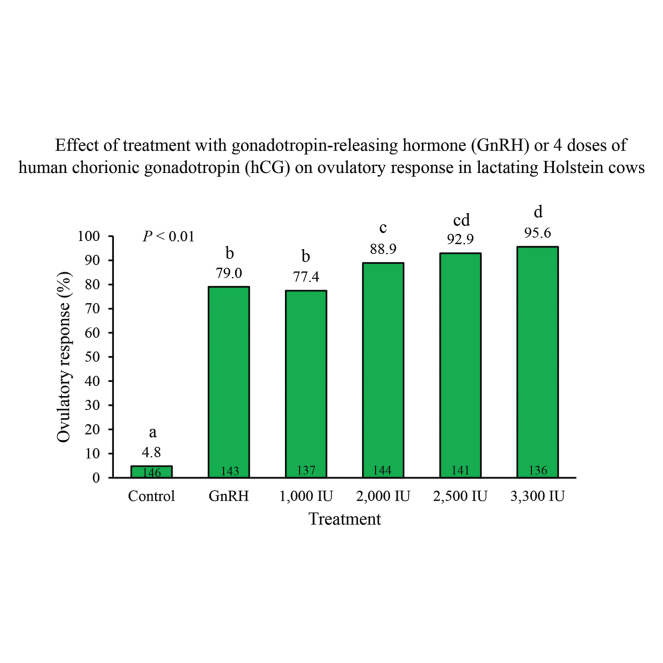
**Summary:** Treatment of lactating Holstein cows with 100 μg of GnRH or 1,000 IU of human chorionic gonadotropin (hCG) 7 d after the last GnRH treatment of a Double-Ovsynch or a Resynch protocol had similar ovulatory responses, whereas cows treated with 2,500 or 3,300 IU hCG had the greatest ovulatory responses. Ovulatory response of cows treated with 2,000 IU or 2,500 IU hCG did not differ, whereas ovulatory response after 3,300 IU was greater than ovulatory response after 2,000 IU hCG.

Human chorionic gonadotropin (**hCG**) is a glycoprotein hormone produced by human embryos during the first days after fertilization and later by the placental syncytiotrophoblast cells (Stenman et al., 2006; de Medeiros and Norman, 2009). Because the β-subunit homology of hCG and bovine luteinizing hormone (**LH**) is ~80% (Pierce and Parsons, 1981), hCG is a potent LH agonist and can induce ovulation of a dominant follicle in cows and heifers (Rajamahendran and Sianangama, 1992), leading to subsequent increases in progesterone (**P4**) concentrations. Inducing ovulation during the early luteal phase with hCG should be more effective than using GnRH because high concentrations of P4 (>3 ng/mL) inhibit the GnRH-induced LH surge that is necessary to induce ovulation (Giordano et al., 2012). In contrast, hCG acts independently of the hypophysis by binding directly to LH receptors, and it is not affected by serum P4 concentrations at the time of administration (Motta et al., 2020).

Lactating dairy cows have most commonly been treated with doses of hCG that vary between d 5 and 7 of the estrous cycle, when the dominant follicle of the first follicular wave has acquired LH receptors and is capable of ovulating (Ireland and Roche, 1982). To the best of our knowledge, no studies have reported the optimal dose of hCG required to induce ovulation of a dominant follicle on d 7 of the estrous cycle in lactating Holstein cows. Typically, hCG doses used in studies have ranged between 1,000 and 3,300 IU (Nascimento et al., 2013a; Besbaci et al., 2020). Therefore, the objective of this study was (1) to conduct a dose-response study for hCG to evaluate the induction of ovulation of a first-wave dominant follicle in lactating Holstein cows in a P4 environment typical for initiation of synchronization protocols such as Ovsynch, and (2) to determine the effect of hCG treatment on subsequent P4 concentrations. We hypothesized that the ovulatory response would increase with increasing hCG dose and would be accompanied by an increase in plasma P4 concentrations 7 d after treatment.

All animal handling and experimental procedures were approved by the Animal Care and Use Committee of the College of Agriculture and Life Sciences at the University of Wisconsin-Madison. This study was conducted from March 2019 to August 2019 on a commercial dairy farm located near Belleville, Wisconsin. During the experimental period, the average number of milking cows was 1,300 and the average daily milk production of the herd was 45 kg/cow per day.

Lactating Holstein cows were submitted to a Double-Ovsynch (**DO**) protocol for first timed AI (**TAI**) as first described by Souza et al. (2008) and modified by Brusveen et al. (2009) after a voluntary waiting period of 73 ± 3 d. Briefly, cows received the first GnRH (100 µg of gonadorelin acetate; Fertagyl; Merck Animal Health, Kenilworth, NJ) treatment of the Pre-Ovsynch portion of the DO protocol followed by treatment with PGF_2α_ (526 µg of cloprostenol sodium; Estrumate; Merck Animal Health) 7 d later and treatment with the second GnRH 3 d later. Cows began the Breeding-Ovsynch portion of the DO protocol 7 d later with GnRH treatment, 2 PGF_2α_ treatments 7 and 8 d later, GnRH 56 h after the first PGF_2α_ treatment, with TAI conducted 16 to 18 h after the last GnRH treatment using conventional semen. Cows failing to conceive to first TAI were submitted to a protocol for resynchronization of ovulation (**Resynch**). The Resynch protocol consisted of GnRH administration to all cows 25 d after TAI. Pregnancy diagnosis was performed 32 d after TAI using transrectal ultrasonography. Nonpregnant cows with a corpus luteum (**CL**) received 2 PGF_2α_ treatments 24 h apart, GnRH 56 h after the first PGF_2α_, and TAI 16 to 18 h later using conventional semen. Nonpregnant cows lacking a CL received an intravaginal P4 controlled internal drug release insert (CIDR; 1.38 g of P4; Eazi-Breed CIDR; Zoetis, New York, NY) and GnRH; 7 d later the CIDR was removed and cows received PGF_2α_, followed by PGF_2α_ 24 h later, GnRH 32 h later, and TAI 16 to 18 h later using conventional semen. Cows diagnosed not pregnant at the pregnancy diagnosis after their initial enrollment into the experiment were eligible to be re-enrolled after re-randomization to treatments. The final database contained 854 observations from 629 cows (211 primiparous, 418 multiparous), of which 486 observations corresponded to a DO protocol (57%), and 368 to a Resynch protocol (43%).

The day of the last GnRH treatment of the DO or Resynch protocols (**G2**) was considered experimental d 0. On d 7, ovarian transrectal ultrasonography was performed using a portable ultrasound machine fitted with a 7.5-MHz linear-array probe (Ibex Pro; E.I. Medical Imaging, Loveland, CO). Cows with at least 1 visible CL ≥15 mm in diameter and 1 follicle ≥10 mm in diameter were blocked by parity (primiparous vs. multiparous) and randomly assigned to receive one of the following treatments: untreated control (**CON**; n = 147), 100 µg of GnRH (n = 144), or 1,000 (n = 138), 2,000 (n = 144), 2,500 (n = 142), or 3,300 (n = 139) IU of hCG (Chorulon; Merck Animal Health). All doses of hCG used in this experiment were from the same production lot. All hormonal treatments were administered i.m. in the semimembranosus or semitendinosus muscle.

Ovarian ultrasonography was conducted in all cows on d 7, and a map of each ovary was drawn, with the location and diameter of the existing CL and all follicles >5 mm, which allowed for determination of the dominant follicle and evaluation of ovulatory response to treatments. Ovarian ultrasonography was performed again in all cows on d 14 to assess ovulatory response to treatment and to record the size of the existing CL and the size and location of the accessory CL. A cow was considered to have ovulated in response to treatment based on the presence of a new CL 7 d after treatment. Follicles and CL were assumed to be spherical to estimate volume. Two perpendicular diameters of the follicles and CL were determined using the on-screen background gridlines, comprising squares with 10-mm sides on the eyepiece of the ultrasound machine. Volume (V) of follicles and CL were calculated as follows: V = (4/3) × βpi; × R^3^, where R = (W/2 + H/2)/2, βpi; = 3.14159, W = largest width, and H = largest height of structure. For all CL in which fluid cavities were detected, the volume of the cavity was calculated and subtracted from the volume of the entire CL.

Blood samples (8 to 9 mL) were collected from all cows on d 7 before treatments were administered and 7 d later on d 14 via puncture of the medial caudal vein or artery using evacuated K_2_-EDTA collection tubes (Vacutainer; BD, Franklin Lakes, NJ). Blood plasma were immediately refrigerated upon collection and were centrifuged (2,000 × *g*, 4°C) for 20 min. Plasma was harvested and stored at −20°C. Plasma P4 concentrations were assayed using a commercial solid-phase RIA kit containing antibody-coated tubes and ^125^I-labeled P4 (ImmuChem Coated Tube Progesterone ^125^I RIA Kit, MP Biomedicals, Costa Mesa, CA) validated for use in bovines (Garbarino et al., 2004; Skenandore et al., 2017). To assess the precision of the assay, quality control samples were made from a pool of charcoal-stripped bovine serum spiked with P4 to a concentration of 2.0 ng/mL and stored at −20°C in aliquots. Quality control samples were repeated at the beginning, in the middle, and at the end of each assay. Plasma samples were run as singlets across 4 assays. The average sensitivity for the assay was 0.03 ng/mL. The average intra- and interassay coefficients of variation were 3.57 and 6.17%, respectively, based on quality control samples repeated in each assay.

Based on an a priori power calculation (Thrusfield et al., 2001), inclusion of at least 78 cows per treatment allowed for detection of a 15-percentage-point difference in ovulatory response (75 to 90%; 95% confidence; 80% power; one-sided test). Statistical analyses were performed using SAS computational software version 9.4 for Microsoft Windows (SAS Institute Inc., Cary NC). Analyses of binary response data (ovulatory response) was performed by logistic regression using the GLIMMIX procedure. Continuous variables (CL volume, follicular size, and P4 concentrations) were analyzed by ANOVA using the MIXED procedure. Before these analyses, the normality of quantitative variables was assessed using the Shapiro-Wilk statistic and graphical methods obtained with the UNIVARIATE procedure. A significant *P*-value for the variable plasma P4 concentration indicated that data were not normally distributed; therefore, data were log-transformed. The selection of the model that best fit the data for each variable of interest was performed by finding the model with the lowest value for the Akaike information criterion, using a backward elimination procedure that removed all variables with *P* > 0.10 from the model. For all variables, treatment and parity were considered fixed effects and forced into the final model. The initial model included the fixed effects of treatment, parity, service number (1 vs. 2+), existing CL volume on d 7, follicular volume on d 7, P4 concentration on d 7, and treatment × parity interaction. The final models included the fixed effects of treatment, parity, service number, and treatment × parity interaction. A significant difference between levels of a classification variable was declared when *P* ≤ 0.05, whereas differences between *P* > 0.05 and *P* ≤ 0.10 were declared a statistical tendency. Differences between least squares means were compared using the Tukey method to adjust for multiple comparisons. Data are presented as least squares means ± standard error of the mean (LSM ± SEM) and as proportions for continuous and binary outcomes, respectively. For log-transformed data, LSM were back-transformed.

The ovulatory response to treatments differed (*P* < 0.01) among treatments and was 4.8, 79.0, 77.4, 88.9, 92.9, and 95.6% for CON, GnRH, 1,000, 2,000, 2,500, and 3,300 IU hCG treatments, respectively (Figure 1). The main effect of parity (*P* = 0.73), the treatment × parity interaction (*P* = 0.28), and service number (*P* = 0.18) were not significant. These results agree with previous studies. For example, treatment with 100 µg of GnRH on d 5 after TAI resulted in an ovulatory response of 70.5% (Baez et al., 2017). When 1,000 IU of hCG was administered 7 d after TAI, the ovulatory response was 70% (Stevenson and Pulley, 2012). Moreover, ovulatory response after treatment with 2,000 IU of hCG on d 5 after TAI was 78.3% (Nascimento et al., 2013a). Treatment with 3,300 IU of hCG on d 5 resulted in an ovulatory response of 93% (Nascimento et al., 2013b), and the same dose applied once between 4 and 9 d after TAI achieved an ovulatory response of 81.3% (Stevenson et al., 2007). Considering that in the present study, treatments were administered 7 d after G2 (as opposed to 5 d after TAI, as in many studies), a greater ovulatory response would be expected because the dominant follicle would be larger. As the dominant follicle increases in size, granulosa cells acquire a greater number of LH receptors, thereby increasing the ovulatory response to LH/hCG (Ireland and Roche, 1982; Stewart et al., 1996). In addition, Sartori et al. (2001) reported a relationship between follicular size and the response to LH or hCG because the dose necessary to induce ovulation in the majority of 10-mm follicles was 6 times greater (4 vs. 24 mg of LH) than the dose required to induce ovulation of 12-mm follicles.

**Figure 1:**
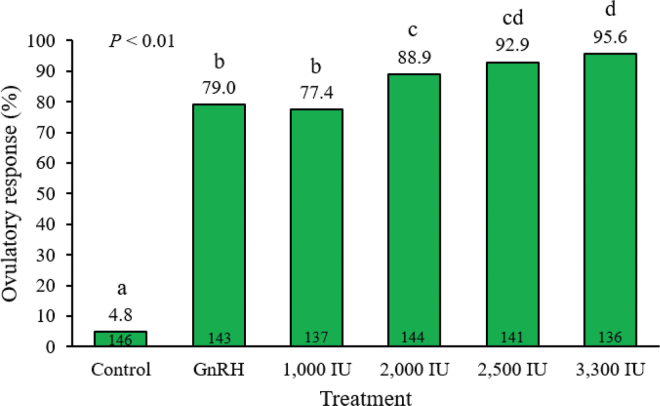
Effect of treatment with GnRH or 4 increasing doses of human chorionic gonadotropin (hCG) on ovulatory response of the first-wave dominant follicle 7 d after the last GnRH treatment of the synchronization protocol (G2) in lactating Holstein cows. Ovulation was determined by the presence of a new corpus luteum in the same location as an ovulatory follicle 7 d after treatment. Proportions with different letters (a–d) differ (*P* ≤ 0.05).

As expected, treatment with GnRH was not as effective as the highest doses of hCG to induce ovulation, although it did not differ compared with 1,000 IU of hCG. Based on data from Giordano et al. (2012), ovulatory response to GnRH decreases when P4 concentrations are high (>3 ng/mL), most likely because of a negative effect of P4 directly on the anterior pituitary to attenuate the preovulatory LH surge. In our study, the mean concentration of P4 on d 7 was 3.34 ± 0.6 ng/mL, constituting a P4 environment that likely attenuated the GnRH-induced LH surge, thereby decreasing the overall ovulatory response. In contrast, the dose-responsiveness of hCG on ovulatory response may be a pharmacological phenomenon, considering that hCG is a potent LH agonist that is not affected by circulating P4 concentrations (Pierce and Parsons, 1981; Rajamahendran and Sianangama, 1992). We speculate that the amount of exogenous hCG administered in conjunction with the hCG half-life in circulation may affect peak circulating concentrations of hCG, which could affect LH receptor binding at the level of the dominant follicle. Further experiments are needed to confirm or reject this idea.

The mean diameter of dominant follicles on d 7 did not differ among treatments (13.6 ± 0.3, 13.7 ± 0.2, 13.7 ± 0.2, 13.4 ± 0.2, 13.4 ± 0.2, and 13.4 ± 0.2 mm for CON, GnRH, 1,000-, 2,000-, 2,500-, and 3,300-IU hCG treatments, respectively; *P* = 0.89) or by parity (13.4 ± 0.2 vs. 13.6 ± 0.1 mm for primiparous vs. multiparous cows, respectively; *P* = 0.12) but differed between cows inseminated after the DO or Resynch protocols (13.8 ± 0.1 vs. 13.3 ± 0.1 mm, respectively; *P* < 0.01). The mean diameter of dominant follicles that ovulated to treatment did not differ (*P* = 0.94) among treatments or by parity (*P* = 0.16) and was greater (*P* < 0.01) than the mean diameter of follicles that failed to ovulate to treatment (13.2 ± 0.1 vs. 12.4 ± 0.1 mm, respectively).

Plasma P4 concentrations did not differ (*P* = 0.85) among treatments on d 7 before treatments were administered (Table 1). Primiparous cows had greater (*P* < 0.01) plasma P4 concentrations on d 7 than multiparous cows (3.7 ± 0.1 vs. 3.2 ± 0.1 ng/mL, respectively). This was expected because multiparous cows generally have higher milk yields, which is associated with a greater DMI and, consequently, increased hepatic P4 metabolism (Sangsritavong et al., 2002; Vasconcelos et al., 2003). There was no difference in plasma P4 concentrations (*P* = 0.18) on d 7 between cows inseminated after the DO versus Resynch protocols (3.3 ± 0.1 vs. 3.4 ± 0.1 ng/mL, respectively), and cows with double ovulation after G2 had greater (*P* < 0.01) plasma P4 concentration than cows with single ovulation (3.8 ± 0.2 vs. 3.3 ± 0.1 ng/mL, respectively).

**Table 1:** Progesterone (P4) concentrations (ng/mL) and corpus luteum (CL) volume (mm^3^) 7 d after the last GnRH treatment of the synchronization protocol (G2), and effect of treatment with GnRH or 4 increasing doses of human chorionic gonadotropin (hCG; 1,000 to 3,300 IU) on mean P4 concentrations and luteal volume 14 d after G2^1^

Item	Treatment^2^						*P*-value
	Control	GnRH	1,000 IU	2,000 IU	2,500 IU	3,300 IU	
Number of cows	146	143	137	144	141	136	
Day 7							
P4	3.7 ± 0.17	3.9 ± 0.17	3.9 ± 0.17	3.8 ± 0.17	3.7 ± 0.17	3.9 ± 0.17	0.85
Existing CL volume	5,767 ± 272	6,955 ± 275	6,305 ± 281	6,644 ± 275	6,734 ± 277	5,974 ± 280	0.27
Day 14							
P4	7.2 ± 0.33^a^	9.8 ± 0.33^bc^	9.6 ± 0.34^b^	10.4 ± 0.33^bc^	10.7 ± 0.34^c^	10.4 ± 0.34^bc^	<0.01
Existing CL volume	9,117 ± 396^a^	9,629 ± 398^ab^	8,918 ± 405^a^	10,666 ± 396^b^	10,357 ± 400^b^	9,949 ± 408^ab^	0.04
Accessory CL volume	—	3,976 ± 275^a^	4,098 ± 280^a^	4,768 ± 274^ab^	5,040 ± 277^b^	5,440 ± 282^b^	<0.01
Total luteal volume	9,117 ± 440^a^	13,605 ± 442^b^	13,017 ± 450^b^	15,434 ± 440^c^	15,397 ± 445^c^	15,389 ± 453^c^	<0.01

a–c Within a row, LSM with different superscripts differ (*P* ≤ 0.05).

1 Data are presented as LSM ± SEM.

2 Seven days after the last GnRH treatment (G2) of a Double-Ovsynch or Resynch protocol, cows were randomly assigned to receive no treatment (control), 100 µg of GnRH, or 1,000, 2,000, 2,500, or 3,300 of IU hCG.

As expected, we observed an increase (*P* < 0.01) in plasma P4 concentrations in all cows from d 7 to 14. The increase in plasma P4 concentrations was greater (*P* < 0.01) for treated than for control cows and differed (*P* = 0.04) for GnRH and 1,000-IU hCG treatments compared with the 2,500-IU hCG treatment (Figure 2). The increase in plasma P4 concentrations is primarily associated with formation of an accessory CL and to the direct luteotropic effect of hCG on the existing CL (Farin et al., 1988; Fricke et al., 1993). When considering treated cows that failed to ovulate, the increase in plasma P4 concentration from d 7 to 14 was greater (*P* < 0.01) than that for CON cows, supporting that gonadotropins have a luteotropic effect on the existing CL. The increase in plasma P4 concentration for cows that failed to ovulate was 3.7 ± 0.3, 5.2 ± 0.6, and 5.0 ± 0.4 ng/mL for CON, GnRH- and hCG-treated cows, respectively (CON vs. GnRH, *P* = 0.02; CON vs. hCG, *P* = 0.01; GnRH vs. hCG, *P* = 0.80).

**Figure 2:**
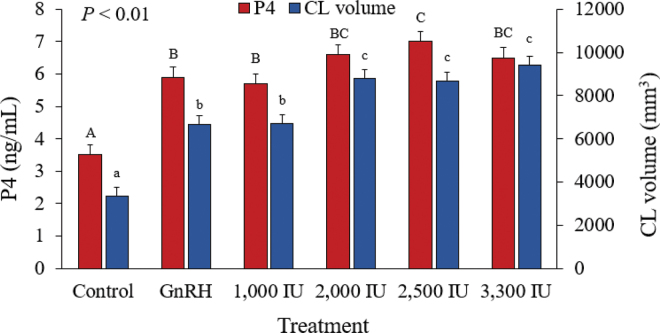
Effect of treatment with GnRH or 4 increasing doses of human chorionic gonadotropin (hCG) on progesterone (P4) concentrations (ng/mL) and total corpus luteum (CL) volume (mm^3^) increase from 7 to 14 d after the last GnRH treatment of the synchronization protocol (G2) in lactating Holstein cows. Data are presented as LSM ± SEM. Uppercase letters (A–C) indicate differences (*P* ≤ 0.05) in mean increase in P4 concentrations; lowercase letters (a–c) indicate differences (*P* ≤ 0.05) in mean increase in total luteal volume (existing CL + accessory CL).

Exogenous hCG binds to LH receptors and mimics the effects of LH by stimulating small luteal cells to increase P4 synthesis. Farin et al. (1988) reported that intravenous treatment with 300 IU of hCG on d 5 and 7.5 of the ovine estrous cycle altered the proportion of small and large luteal cells on d 10, potentially changing total production of P4 by the CL because ~80% of luteal P4 is produced by large luteal cells (Niswender et al., 2000). The increase in P4 concentrations after treatment with gonadotropins on d 5 of the estrous cycle has been consistent among many studies (Santos et al., 2001; Galvão et al., 2006; Nascimento et al., 2013a,b). In a study by Vasconcelos et al. (2011), greater concentrations of P4 were detected on d 14 for gonadotropin-treated cows, with no difference between GnRH and 2,500-IU hCG treatments, in agreement with our results (Table 1). In contrast, Beltran and Vasconcelos (2008) reported that P4 concentration on d 12 was greater for treated than for control cows, and that the increase was greater for cows treated with 2,500 IU of hCG than for cows treated with GnRH.

Luteal volume on d 7 did not differ (*P* = 0.27) among treatments (Table 1). Luteal volume did not differ (*P* = 0.84) between parities on d 7 when cows with double ovulations to G2 were removed from the analysis (5,819.2 ± 183 vs. 5,906.1 ± 135 mm^3^ for primiparous and multiparous cows, respectively). When cows that double ovulated to G2 were included in the analysis, luteal volume differed (*P* = 0.05) between parities on d 7 (6,067.4 ± 200 vs. 6,552.5 ± 138 mm^3^ for primiparous and multiparous, respectively). This difference was likely due to the greater (*P* < 0.01) incidence of double ovulations to G2 observed in multiparous cows (8.0%; 22/274 vs. 20.0%; 116/580 for primiparous and multiparous, respectively). As reported by Fricke and Wiltbank (1999), multiparous and high-producing cows generally have an increased rate of double ovulations because high feed intake increases hepatic steroid metabolism, which alters the endocrine environment sufficiently to allow for deviation of 2 follicles during selection of a dominant follicle. Finally, luteal volume did not differ (*P* = 0.52) on d 7 between cows submitted to DO compared with Resynch protocols (6,247.3 ± 151 vs. 6,594.5 ± 173 mm^3^, respectively).

The volume of the existing CL increased (*P* < 0.01) from d 7 to 14 in all cows. There was an effect (*P* = 0.04) of treatment on volume of the existing CL on d 14 (Table 1). In contrast to our results, Nascimento et al. (2013a) reported that treatment with 2,000 IU of hCG 5 d after TAI did not affect the volume of the existing CL. Stevenson et al. (2007) reported that the existing CL volume increased in cows treated with 3,300 IU of hCG but tended to decrease for cows treated with GnRH compared with untreated controls. In the latter study, total luteal volume (existing CL + accessory CL) was increased in hCG-treated cows compared with control cows, and the same finding was reported by the same author in a subsequent study (Stevenson and Pulley, 2012). The increase in total luteal volume from d 7 to 14 in the present study differed (*P* < 0.01) among treatments (Table 1, Figure 2) because of the effect of treatment on the formation of an accessory CL and the luteotropic effect on the existing CL.

In conclusion, doses of hCG ≥2,000 IU resulted in a greater ovulatory response in lactating Holstein cows than 100 µg of GnRH or 1,000 IU of hCG. We consider 2,500 IU of hCG to be the optimal dose to induce ovulation 7 d after G2 because it did not differ from the ovulatory response achieved with 3,300 IU, and it resulted in the greatest numerical increase in plasma P4 concentration between d 7 and 14. Data on the optimal dose of hCG to induce ovulation 7 d after induction of ovulation will be useful for the design of future studies on the effects of hCG on the reproductive biology of lactating dairy cows.
